# Valorization of Pineapple Crown for Carboxymethylcellulose Production: Optimization of Pulping Processes, Structural Characterization, and Potential as Seed Coating

**DOI:** 10.3390/polym18101216

**Published:** 2026-05-16

**Authors:** Eulina Fernandes Damião, Diego Palmiro Ramirez Ascheri, Itamar Rosa Teixeira, Roberta Signini, Rejane Dias Pereira Mota, José Luis Ramírez Ascheri

**Affiliations:** 1 Programa de Pós-Graduação Stricto Sensu em Engenharia Agrícola (CPPGEA) da Universidade Estadual de Goiás (UEG), Câmpus Central—Sede: Anápolis—CET Henrique Santillo, BR-153, nº 3.105, Fazenda Barreiro do Meio, Anápolis CEP 75132-903, GO, Brazil; eulina@aluno.ueg.br (E.F.D.); itamar.texeira@ueg.br (I.R.T.); 2 Programa de Pós-Graduação Stricto Sensu em Ciências Moleculares (PPGCM) da Universidade Estadual de Goiás (UEG), Anápolis CEP 75132-903, GO, Brazil; roberta.signini@ueg.br; 3 Instituto Federal de Goiás, Campus Anápolis, Anápolis CEP 75131-457, GO, Brazil; rejane.mota@ifg.edu.br; 4 Food Extrusion Pilot Plant and Cereal Quality Lab., Embrapa Agroindústria de Alimentos, Av. das Américas, 29501-Guaratiba, Rio de Janeiro CEP 23020-470, RJ, Brazil; jose.ascheri@embrapa.br

**Keywords:** *Ananas comosus*, *Phaseolus vulgaris*, *Bacillus subtilis*, carboxymethylcellulose, physiological seed quality, agrobiodiversity, sustainability, pineapple crown, seed coating, seed germination

## Abstract

The increasing demand for sustainable agricultural inputs has driven interest in biodegradable polymers from agro-industrial residues. Pineapple crown biomass (PCB), a widely available lignocellulosic waste, represents a promising feedstock for producing carboxymethylcellulose (CMC). However, the optimal pulping and bleaching conditions for CMC synthesis from this residue remain underexplored. Nevertheless, the combination of CMC derived from PCB with *Bacillus subtilis* as a seed coating agent for the bean cultivar has not yet been investigated. Here, we produced cellulosic pulps from PCB using a bioreactor, varying NaOH concentration (1–3%), pulping time (1.5–2.5 h), bleaching volume (55–75 mL) and time (60–120 min). The selected pulping condition (2% NaOH, 1.5 h) yielded pulp with high purity (83.9%) and crystallinity (76.35%). After bleaching (65 mL, 90 min), the material was suitable for CMC synthesis under two conditions: CMC1 and CMC2. CMC2 showed a higher degree of substitution (1.010) than CMC1 (0.620) but led to reduced seed germination (77.67%) due to excessive water retention and fungal growth. In contrast, CMC1, with or without *B. subtilis*, maintained high germination (91%) and significantly increased seedling length (21.30 cm). We conclude that PCB is a viable feedstock for CMC production, and CMC1 exhibits strong potential as an effective seed coating agent for sustainable agriculture.

## 1. Introduction

In agriculture, the pineapple production chain generates residual biomass (roots, stems, and leaves), which is commonly discarded or incorporated into the soil [1]. Fruit processing also generates by-products such as peel, core, and crown. In 2024, Brazil—with an estimated national production of approximately 1.48 billion “Pérola” pineapple fruits [2]—likely generated between 200,000 and 500,000 metric ton of crowns alone, assuming an average fruit mass of 0.9 to 1.8 kg [3] and a crown proportion of 10–25% per fruit [4]. The frequent disposal of this residue in open dumps represents an environmental liability and a loss of potentially valuable biomass.

The pineapple crown has been investigated for multiple applications, including use in bio-adsorption systems for water treatment [5] and natural gas purification [6], production of proteolytic enzymes [7], medicinal applications [8], and as a raw material for nutritional, pharmaceutical, and textile applications, as well as for the production of bioactive compounds and biofuels [5,9,10].

This residue, composed primarily of cellulose (36–83%), hemicellulose (16–19%), and lignin (5–15%) [11,12,13], is a promising feedstock to produce cellulosic pulps and their derivatives. Such derivatives—particularly those used to formulate bioslurries for phytosanitary control or as coating agents in organic agriculture [14,15,16,17]—have strong potential to enhance plant performance and productivity, whether applied alone or in combination with growth-promoting and biological control agents [18,19,20].

The chemical conversion of cellulose requires treatments capable of removing hemicellulose, lignin, and chromophoric compounds without compromising the chemical structure of the biopolymer. Variables such as alkali concentration, bleaching agents, temperature, and reaction time must be systematically evaluated to obtain high-quality cellulose that can be readily derivatized into water-soluble polymers such as carboxymethylcellulose (CMC) [21,22,23].

The selection of the most suitable treatment for producing the unbleached pulp intended for subsequent bleaching must be carefully assessed, considering not only the Kappa number (*κ*) and structural characterization but also product quality and process sustainability. Different combinations of factors may lead to similar outcomes, and experimental design should identify the conditions that best balance efficiency and environmental impact [24].

In this context, for pineapple leaf pulping, active alkali charges between 18% and 24% and increasing alkali concentration reduce the *κ* from 22.0 to 15.5, indicating greater de-lignification, but also decrease pulp yield from 32.9% to 29.5% [25]. This behavior shows that harsher conditions, although effective at lignin removal, can compromise productivity and cellulose integrity, reinforcing the need for careful optimization.

Corroborating these findings, preliminary studies on pineapple crown have shown that a mild treatment (in 2% (*w*/*v*) NaOH for 1.5 h (h)) removes 83.9% of lignin, yielding pulp with a *κ* of 8.3. In contrast, harsher conditions (in 3% (*w*/*v*) NaOH for 2.5 h) promote only slightly higher lignin removal (84.3%; *κ* = 8.1) but lead to increased recondensation of residual lignin and greater cellulose loss [26]. These results indicate that increasing alkaline treatment severity does not necessarily result in proportional gains in delignification and may, conversely, impair pulp quality and process efficiency.

The unbleached pulp, however, still contains residual lignin and chromophoric compounds that impart a brown color. To obtain a high-value product such as CMC, a bleaching step is essential. This process aims to remove these components, resulting in a purified, white cellulose pulp [27]. For this purpose, oxidizing agents are commonly used, such as sodium chlorite solutions [28], mixtures of hydrogen peroxide and glacial acetic acid [29], and hydrogen peroxide combined with sodium hydroxide [30,31], with efficiency optimized by controlling process variables such as time, temperature, and reagent concentration.

The bleached pulp must exhibit high cellulosic purity, as indicated by the absence of residual lignin and hemicellulose in spectroscopic analyses; high thermal stability with low char residue, as determined by thermogravimetric analysis; and a high crystallinity index, determined by X-ray diffraction (XRD). XRD enables evaluation of the structural organization of cellulose, a key parameter for the efficiency of the carboxymethylation reaction. Proper interpretation of diffractograms is essential for accurately determining the crystallinity index (CrI) and understanding structural modifications induced by the treatments [32].

The CrI and the full width at half maximum of diffraction peaks reflect cellulose purity, chemical accessibility, and reactivity, guiding the selection of conditions that preserve the crystalline integrity of the biopolymer. These parameters are therefore widely used to optimize the pulp bleaching process and are essential for subsequent application in CMC synthesis [33].

CMC, a renewable and biodegradable cellulosic derivative, has emerged as a strategic bio-input in agriculture. Its use is favored by excellent solubility and dispersibility, which facilitate uniform application and improve the mobility of inputs. It also acts as an encapsulating agent, extending the shelf life of bioactive compounds and reducing the need for reapplication [16].

Beyond agriculture, CMC has attracted interest for intelligent packaging for the controlled release of active compounds [34,35,36] and as a seed coating agent, promoting adhesion of chemical or microbiological products [37,38]. However, its effectiveness as a primary coating material remains underexplored [20]. The effectiveness of these functionalities depends directly on parameters such as purity, degree of polymerization, and degree of substitution (DS), allowing precise tuning for specific agricultural demands. Therefore, the DS and thermal stability of CMC must be evaluated to select the most suitable material as a coating agent.

Fourier transform infrared spectroscopy (FTIR) is an effective tool for estimating DS, as the intensity of the characteristic carboxymethyl group (COO^−^) band at 1600 cm^−1^ is directly related to the number of substituent groups incorporated into the cellulose chain [39,40]. Additionally, thermogravimetric analysis (TGA) enables evaluation of thermal stability, an essential parameter to ensure that the coating withstands drying and storage without degradation, and provides insight into carboxymethyl group content through the char residue formed during pyrolysis [41]. Thus, combining FTIR and TGA enables comprehensive characterization of CMC, guiding the selection of the most suitable material for specific applications such as seed coating.

This gap is particularly relevant for the bean cultivar BRS FC104, developed by Embrapa, which is super-early (cycle < 65 days) and combines high yield potential with resistance to common mosaic and moderate resistance to major diseases [42]. Common bean occupies a strategic position in Brazilian agriculture, being widely cultivated and consumed [43]. The adoption of sustainable technologies, such as seed coating with biopolymers and beneficial microorganisms, is fundamental to increasing productivity and agronomic efficiency.

*Bacillus subtilis* has been consolidated as a promising agent for seed treatment and coating. This microorganism stands out for its ability to produce secondary metabolites, form spores, withstand adverse conditions, and promote benefits related to germination, vigor, and initial seedling establishment [44,45]. Studies conducted on bean crops indicate that inoculation with *B. subtilis* can result in significant increases in growth variables and productivity, including increased seedling dry weight and improved initial physiological performance [46,47].

Nevertheless, the combination of CMC derived from agro-industrial residues (pineapple crowns) with *B. subtilis* as a seed coating agent for the BRS FC104 bean cultivar has not yet been investigated.

In this context, this study aimed to optimize the production of cellulosic pulp from pineapple crown using an experimental design in a bioreactor (70 °C, 700 rpm), synthesize CMC from the bleached pulp, characterize the materials by *κ*, FTIR, XRD, and TGA, and evaluate the potential of CMC, alone or associated with *B. subtilis*, as a coating agent for common bean seeds, contributing to the sustainable use of agro-industrial residues.

## 2. Materials and Methods

### 2.1. Material

Pineapple crown biomass (PCB) of the Pérola variety was provided by Fazenda Cachoeira (Jaraguá, Brazil). Healthy crown leaves were dehydrated in an oven with forced air circulation (Marconi, MA-035, Piracicaba, Brazil) at 80 °C until constant mass, followed by grinding in a Willy SPlabor mill (Mod. SP–31, Pres. Prudente, Brazil) to a particle size ≤ 1 mm.

The reagents sodium chlorite (NaClO_2_, ≥80%), sodium hydroxide (NaOH, ≥97%), acetic anhydride ((CH_3_CO)_2_O, ≥98.5%), glacial acetic acid (CH_3_COOH, ≥99.7%), chloroacetic acid (C_2_H_3_ClO_2_, ≥98.5%) and isoamyl alcohol ((CH_3_)_2_CHCH_2_CH_2_OH, ≥99.7%) were acquired from Sigma-Aldrich (St. Louis, Mo, USA). All other reagents were of analytical grade and were employed without additional purification.

### 2.2. Pulping Process of Pineapple Crown Biomass

The unbleached pulp (Ubp) was extracted by applying different sodium hydroxide (NaOH) concentrations (C1) and reaction times (t1) [20], in a completely randomized design in a 3^2^ factorial arrangement (C1 = 1.0%, 2.0%, and 3.0%, *w*/*w*, and t1 = 1.5, 2.0, and 2.5 h), in triplicate. Pulping was conducted in a bioreactor (Mod. TEC-BIO-FLEX, Piracicaba, Brazil) with a PCB/C1 ratio of 1/20 (*w*/*v*), at 70 °C and stirring at 700 rpm. After extraction, the Ubp was filtered through cheesecloth and washed with abundant distilled water until the pH was neutral, then dried in an oven at 45 °C for 12 h.

### 2.3. Bleaching Process of Unbleached Pulp

Bleaching was performed using a clarifying solution consisting of 1.35% NaOH, 3.75% CH_3_COOH and 1% NaClO_2_ [48]. The experimental design was carried out in a 3^2^ factorial arrangement, varying the volume of the clarifying solution relative to 1.0 g of Ubp (V: 55 mL, 65 mL, and 75 mL), and the reaction time (t2 = 60 min, 90 min, and 120 min). Bleaching was conducted in a TEC-BIO-FLEX bioreactor at 70 °C/700 rpm. The bleached pulp (BP) was filtered through cheesecloth and washed with abundant distilled water until the pH was neutral, followed by drying at 45 °C for 12 h.

### 2.4. Synthesis of Carboxymethylcellulose

CMC was obtained by alkalization and etherification of BP [22]. The selected BP was divided into two samples, S1 and S2, each added with 180 mL of isopropyl alcohol (1:30, *w*/*v*) and stirred for 60 min. Then, the samples were added with 30% NaOH (for S1) and 50% NaOH (for S2) until a yellowish color change occurred, with continuous stirring for an additional 60 min.

In the etherification step, the samples were acidified with 5 and 7 g of chloroacetic acid, respectively. The process was conducted in a bioreactor at 700 rpm, 60 °C, for 2.0 h and 2.5 h for S1 and S2, respectively. At the end of the reaction, the CMCs were filtered and added with 400 mL methyl alcohol, under stirring for 40 min, followed by neutralization with CH_3_COOH until reaching pH 7 and drying at 50 °C until constant mass, obtaining samples CMC1 and CMC2, respectively.

### 2.5. Effect of CMC Associated with B. subtilis on Common Bean Seed Coating

CMC at 2% (*w*/*v*) solutions was used as a base for coating common bean seeds [49] and the *B. subtilis* strain Guentai C3102 (10^10^ CFU/g) was used in the amount of 0.425 g for every 300 seeds. The experimental design was completely randomized, comprising six treatments: control (uncoated), CMC1, CMC2, *B. subtilis* (BS), CMC1 associated with *B. subtilis* (C1BS), and CMC2 associated with *B. subtilis* (C2BS). Each experimental unit consisted of a 150 mL beaker containing 300 common bean seeds (cultivar BRS FC104), which were immersed in the coating solution for 5 min and then air-dried for 24 h.

Seed physiological quality was evaluated by germination (GER) and first count (FC) tests, conducted at 25 °C for 8 days. For this purpose, 50 seeds per treatment were placed to germinate on Germitest paper previously moistened to 2.5 times the mass of the dry paper. FC was evaluated on the 5th day and GER on the 8th day [50].

Seedling dry weight (SDW) and seedling length (SL) were evaluated after 10 days of growth in the dark at 25 °C. Primary root length was measured in normal seedlings, and dry weight was determined after drying in an oven at 80 °C for 24 h [51].

### 2.6. Analytical Characterization

#### 2.6.1. Determination of Kappa Number and Calculation of Lignin Extraction Efficiency

*κ* was determined according to procedure T236 om-06 [52]. The methodology is based on the oxidation of residual lignin in Ubp by potassium permanganate (KMnO_4_) in an acidic medium, followed by iodometric titration with sodium thiosulfate (Na_2_S_2_O_3_). E*κ* was calculated based on the *κ* value of the PCB (*κ*_PCB_) as:

(1) $$E\kappa =100\frac{\left(\kappa_{PCB}-\kappa_{Ubp}\right)}{\kappa_{PCB}}$$

#### 2.6.2. FTIR Spectrophotometric Analysis

A Perkin Elmer infrared spectrophotometer (Model Spectrum Frontier FT-IR/NIR; Perkin Elmer, Norwalk, CT, USA) was used, in the spectral region between 4000 and 400 cm^−1^, with a resolution of 4 cm^−1^. The samples were previously dried in an oven at 60 ± 1 °C for 12 h, crushed, and mixed with KBr in a 1/100 (*w*/*w*) ratio. The peak intensity of the main functional groups of cellulose, hemicellulose, and lignin, as well as the main functional groups of lignin degradation or recondensation present in the samples, were determined [22].

#### 2.6.3. X-Ray Diffractometry

X-ray diffraction analysis of the samples was performed on a Bruker diffractometer (D6 PHASER, Bruker Corporation, Billerica, MA, USA), following the methodology of the instrument’s manual, with CuKα radiation (λ = 1.5406 Å), at 25 °C. The diffraction scan range was defined at angles 5 < 2θ < 50° with a scan rate of 4.12°/min. The operating voltage was 20 kV and the current was 40 mA.

The spectra were subjected to smoothing and baseline correction for the measurement of relevant peak intensities. The CrI was calculated using the deconvolution method [53], in which the areas of the crystalline and amorphous peaks were determined by fitting with a Gaussian function, according to the following equation:

(2) $$\mathrm{CrI}\;\left(\%\right)=100\;\times \;\frac{A_{c}}{A_{\;C}+{\;A\;}_{A}}\;\;\;\;\;\;\;\;\;\;\;\;\;\;\;\;\;\;\;\;\;\;\;\;\;$$

where A_C_ is the sum of the areas of the crystalline peaks corresponding to the planes (110), (110), (200), and (004) at 2θ ≈ 14.6°, 16.6°, 22.5°, and 34.5°, respectively. A_A_ is the amorphous area (centered at approximately 18° 2θ), calculated from the diffractograms.

#### 2.6.4. Thermogravimetric (TGA) and Differential (DTGA) Analysis

Thermal analysis was performed using a Pyris 1 TGA (Thermogravimetric Analyzer) from PerkinElmer (Shelton, CT, USA). Between 4 and 5 mg of each sample was placed in a platinum sample holder and submitted to heating at 10 °C per min^−1^ in a nitrogen atmosphere with a flow of 20 mL min^−1^ up to 700 °C. For each event, the following parameters were determined: initial temperature of mass loss (Ti), which represents the point at which the DTG curve begins to deviate from the baseline; final temperature of mass loss (Tf), which represents the point at which the DTG curve returns to its baseline; temperature of maximum mass loss rate on the DTG curve (TDTG); mass loss at each stage (Δm), which represents the percentage of mass lost from the initial temperature to the final temperature on the TG curve; carbonization residue (RC) [48].

### 2.7. Statistical Analysis

A second-order polynomial model was applied to the *κ* and CrI results of the Ubp and BP, respectively [54]:

Y = β_0_ + β_1_X_1_ + β_2_X_1_^2^ + β_3_X_2_ + β_4_X_2_^2^ + β_5_X_1_X_2_ + β_6_X_1_X_2_^2^ + β_7_ X_1_^2^X_2_ + β_8_X_1_^2^X_2_^2^ + *ε* (3)

where X_1_ and X_2_ are the variables related to factors C1 and V (for X_1_) and t1 and t2 (for X_2_); β0 is the intercept point; β_1_ to β_8_ refer to the linear, quadratic, and two-way interaction effects, as applicable; *ε* = experimental error.

Model selection was performed using analysis of variance (ANOVA) and the adjusted coefficient of determination ($R_{adj.}^{2}$). Regression coefficients were estimated by the least squares method and probability value (*p* < 0.05).

The best condition of the bleaching process was defined using the optimization algorithm [55], with the desirability function (D) restricted to the interval [0, 1], for which the lower, medium, and upper limits were set at 0, 0.5, and 1.0, respectively. If the response is as desired, D = 1, and if the response is outside the acceptable region, D = 0.

The seed quality test responses were subjected to ANOVA to verify significant differences among treatments, adopting a 5% probability level. When significant differences were detected, Tukey’s test was applied for multiple comparison between pairs of means of the three treatments, maintaining the significance level (*p* < 0.05).

Statistical analyses were generated using Statistica 14.1.0.8 software [56], and graphs were generated using OriginPro 2026 [57].

## 3. Results and Discussion

### 3.1. Chemical and Structural Analysis of Biomass and Unbleached Pulps

#### 3.1.1. Evaluation of Kappa Number and Lignin Extraction Efficiency of Ubp Treatments

The *κ* values ranged from 8.1 to 21.8 (Table 1), being lower than that of PCB (51.5 ± 2.2). The E*κ* values were higher than 57%, with emphasis on Ubp4 (83.9%), Ubp8 (81.6%) and Ubp9 (84.3%), the latter being the most efficient, possibly due to the higher C1 and t1 (3% NaOH/2.5 h). However, Ubp4 was practically equal (E*κ* = 83.9%) under moderate conditions (2% NaOH/1.5 h), probably due to saturation in the delignification process, in which, above a plateau of approximately 84% lignin removal, increases in C1 and t1 do not result in increased delignification of PCB.

The ANOVA (Table 2) revealed that the polynomial model of Equation (3) fitted the *κ* values well, showing interaction (C1_LQ_ × t1_LQ_, *F* = 61.7, *p* < 0.001). This indicates that de-lignification efficiency depends not only on the levels of each factor but also on their synergistic action.

The polynomial regression adequately fitted the *κ* values with $R_{adj.}^{2}$ = 0.958, indica-ting that more than 95% of the observed variation was explained by the adopted model. The CR_Ubp_ related to t1^2^, C1 × t1^2^, and C1^2^ × t1^2^ were not statistically significant (*p* > 0.05). The model equation is expressed as:

*κ_Ubp_* = 216.39 − 216.95 C1 + 55.19 C1^2^ − 142.64 t1 + 149.26 C1 × t1 − 38.94 C1^2^ × t1 (4)

**Figure 1:**
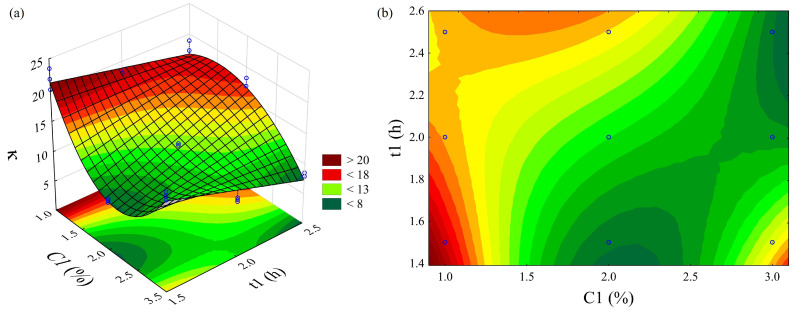
Kappa number (*κ*) values of unbleached pulp from pineapple crown biomass as a function of NaOH concentration (C1) and reaction time (t1). (**a**) Response surface plot and (**b**) contour plot.

The negative linear coefficient (C1 = −216.95) combined with the positive quadratic coefficient (C1^2^ = 55.19) produces an upward-concave curvature, evidencing at least two minimum regions for *κ* (Figure 1a), corresponding to the Ubp4 and Ubp9 treatments, as previously mentioned. The contour plot (Figure 1b) reinforces the saturation hypothesis in the process, revealing a wide minimum region that encompasses different combinations of C1 and t1, such as 1.5% NaOH/1.5 h, 2.0% NaOH/2.0 h, and 3.0% NaOH/2.0 h. In this region, the lowest experimentally obtained *κ* values are concentrated: 8.3 (Ubp4), 9.5 (Ubp8), and 8.1 (Ubp9), indicating that moderate conditions are as effective as harsher conditions for lignin removal.

This behavior suggests that delignification reaches a plateau beyond certain conditions, indicating that additional increases in C1 or t1 do not result in significant further delignification but may imply higher input consumption and possible carbohydrate degradation.

#### 3.1.2. Fourier Transform Infrared Spectroscopy Analysis of Ubp Samples

**Figure 2:**
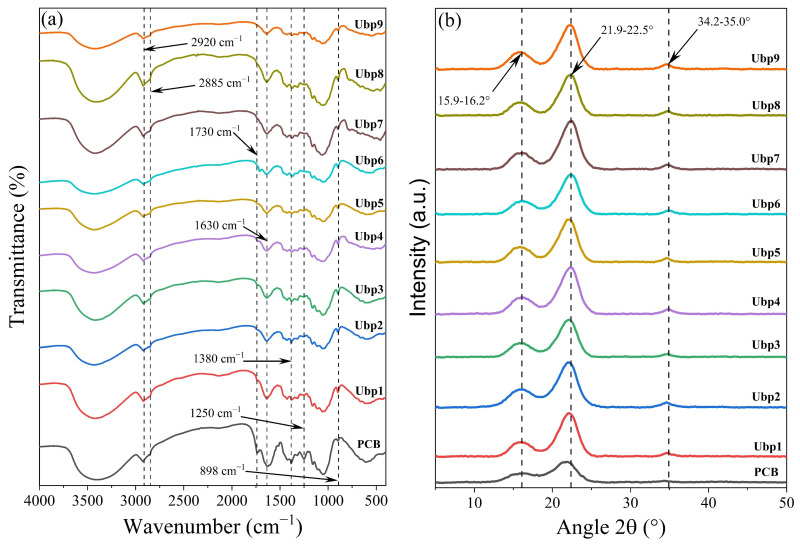
FTIR and XRD analysis of unbleached pulps (Ubp1 to Ubp9) from pineapple crown biomass (PCB) as a function of NaOH concentration and reaction time. (**a**) FTIR spectra and (**b**) XRD patterns.

The bands at 2920 and 2885 cm^−1^ (Figure 2a) correspond, respectively, to the asymmetric and symmetric stretching of C-H bonds present in lignin and hemicellulose [11,58]. After alkaline treatment, the I_2885_ values approached zero, while the I_2920_ values showed a significant increase for Ubp3, Ubp7, and Ubp8 (Table 3). Comparing these results with the *κ* values, it is observed that in Ubp8 (with the lowest *κ*) the I_2920_ = 4.43 is higher than that of Ubp4 (I_2920_ = 2.72) and Ubp9 (I_2920_ = 2.05). This discrepancy suggests that, in Ubp8, lignin may have condensed, stabilizing aliphatic groups in the fibrous matrix [59,60,61], without compromising E*κ*.

Except for Ubp6, the reduction in I_1720_ (C=O stretching of acetyl ester groups or carboxylic ester groups of lignin [62]) in the other treatments corroborates the removal of hemicellulose, reaffirming the efficiency of the alkaline process, a behavior also observed in cellulose extracted from pineapple crown treated with alkali [11,26]. This decrease indicates the effective removal of amorphous components from PCB, reflecting a higher degree of purification and relative crystallinity of the cellulosic fraction in the Ubps.

The band at 1630 cm^−1^ is attributed to the angular deformation of adsorbed water (H–O–H) in the lignocellulosic matrix [63,64]. The reduction in I_1630_ in Ubps (between 3.7 and 9.4), compared to PCB (11.77), indicates a decrease in matrix hydrophilicity. This change is consistent with the removal of hemicellulose (hydrophilic) and with modifications to the fiber surface by the alkaline process. The persistence of a signal in this region suggests that water retention occurs. This may be related to the formation of a porous structure during treatment or to changes in recondensed residual lignin [60,65,66,67].

Thus, I_1720_ remains the direct marker of hemicellulose removal, while variations in I_1630_ reflect changes in raw pulp hydrophilicity, which are an indirect but important consequence of the chemical rearrangements promoted by the treatment, including the possible stabilization of aliphatic groups evidenced by the bands at 2920 cm^−1^.

The reduction in hydrophilicity, evidenced by the decrease in the band at 1630 cm^−1^, was more pronounced in Ubp9 (3.7) and Ubp6 (4.36), while Ubp4 (5.92) and Ubp8 (7.11) showed intermediate values. Considering that Ubp4, Ubp8, and Ubp9 have similar *κ* values (8.1–9.5), these differences in water retention indicate that, although equally delignified, the pulps have distinct surface properties. The higher intensity in Ubp8 suggests that the possible condensed lignin in this sample may form a more porous matrix or one with greater affinity for water, while Ubp9, subjected to harsher conditions, appears to result in a more hydrophobic surface.

The bands located between 1250 cm^−1^ and 1380 cm^−1^, corresponding to the symmetric C–O stretching of hemicellulose and lignin [68,69] and to the symmetric in-plane angular deformation of cellulose, hemicellulose, and lignin [64,65], respectively, assisted in evaluating the integrity of the lignin structure. This behavior is a direct marker of β-O-4 ether bond cleavage and PCB delignification by the alkaline process.

The systematic increase in I_1380_, associated with the deformation of –CH_3_ groups [70], was observed in all treated samples, but with variable magnitudes. When overlaid with *κ* data, this behavior reinforces the idea that delignification efficiency (low *κ*) is not necessarily correlated with the complete elimination of aliphatic groups. Ubp4, for example, has a *κ* of 8.3 and I_1380_ = 1.69, while Ubp8 (*κ* = 9.5) has I_1380_ = 1.65, practically identical values. This suggests that, at the minimum points of the response surface (Figure 1a), the residual chemical composition may be similar, even starting from different operating conditions.

The band at 898 cm^−1^, associated with the symmetric stretching of the β(1→4) glycosidic bond typical of cellulose [64,69], showed higher intensity in the treated samples compared to PCB. This behavior indicates that, with the selective removal of non-cellulosic components, the spectral contribution of the cellulosic fraction becomes more prominent, resulting in a material with higher relative cellulose purity [11,58,71]. I_898_ was higher in Ubp4 (3.62) and Ubp9 (2.85), which also have the lowest *κ* values. This correlation indicates that, in treatments that reached the response surface minima, the removal of non-cellulosic components was sufficiently effective to enrich the cellulosic fraction, conferring greater relative purity to the pulp. However, Ubp8, although also with low *κ*, showed intermediate intensity (2.44) in this band, suggesting that the gain in cellulosic purity may not be linearly proportional to *κ* reduction when lignin condensation phenomena occur.

#### 3.1.3. X-Ray Diffraction Analysis of Ubp Samples

DRX shows the semi-crystalline nature of PCB (Figure 2b), exhibiting the characteristic profile of cellulose type I, with peaks at 16°, 22°, and 34° (2θ), attributed, respectively, to the (110), (200), and (004) planes of its crystalline structure [72,73], results consistent with those reported in the literature for pineapple leaf [74,75].

High alkaline conditions can alter the cellulose polymorph, as observed in pineapple leaves treated with 5% NaOH, 90 °C, 24 h, which induced the transition to cellulose type II, evidenced by peaks at 12.1°, 20.2°, and 22.0° (2θ) [76]. The absence of these peaks in the diffractograms of this work indicates that there was no phase change during pulping, preserving cellulose type I (Figure 2b).

The significant increase in I_002_ after alkalization indicated an increase in the crystalline cellulosic fraction. The results ranged 70.81 ≤ I_(200)_ ≤ 92.18 a.u., 1.8 to 2.3 times higher than that of PCB (I_(200)_ = 39.29 a.u.) (Table 3).

Compared to PCB (CrI_Ubp_ = 59.30%), the other treatments showed high values of 64.69% ≤ CrI_Ubp_ ≤ 89.25% (Table 3). Ubp2 and Ubp8 achieved the lowest and highest indices (64.69% and 89.25%, respectively), indicating that higher-impact treatments may not only remove hemicellulose but also promote lignin recondensation or affect less ordered regions of cellulose [11,26], limiting the increase in crystallinity.

Except for Ubp2 (CrI_Ubp_ = 64.69%), the other treatments achieved high CrI_Ubp_ values ≥ 75.43% (Table 3), reaching an ideal relationship between purification and preservation of the crystalline structure. The CrIs of the samples were higher than the CrI (~68%) reported for pineapple crown cellulose under more severe conditions (10% NaOH/5 h) [11]. Specifically, Ubp1, Ubp5, and Ubp7 to Ubp9 (79.53% ≤ CrI_Ubp_ ≤ 89.25%) exceeded the value obtained for the same material under conditions of 4% NaOH/1 h (CrI_Ubp_ = 77.89%) [26], with Ubp8 (3% NaOH/2 h) reaching a maximum value of 89.25%.

The correlation between delignification and crystallinity was evidenced by the low *κ* and high CrI_Ubp_ values of Ubp4 (8.3; 76.35%), Ubp8 (9.5; 89.25%), and Ubp9 (8.1; 82.17%). However, although Ubp4 has slightly lower crystallinity than Ubp8, FTIR reveals selective extraction, with moderate intensity of the band at 1630 cm^−1^, indicating that residual lignin remains chemically accessible, favoring the bleaching step. In contrast, Ubp8, despite having the highest crystallinity, presents condensed lignin, recalcitrant to bleaching agents. Ubp9, despite the lowest *κ* and highest crystallinity, uses higher impact conditions, with higher input consumption, and is hydrophobic, which may negatively influence subsequent chemical modification steps.

Given the above, the Ubp4 treatment was chosen because it combines high crystallinity (76.35%) and low *κ* (8.3) with the preservation of non-condensed residual lignin accessible to bleaching, under moderate operating conditions (2% NaOH/1.5 h).

### 3.2. Structural Analysis of Bleached Pulp

#### 3.2.1. Fourier Transform Infrared Spectroscopy Analysis of BP Samples

**Figure 3:**
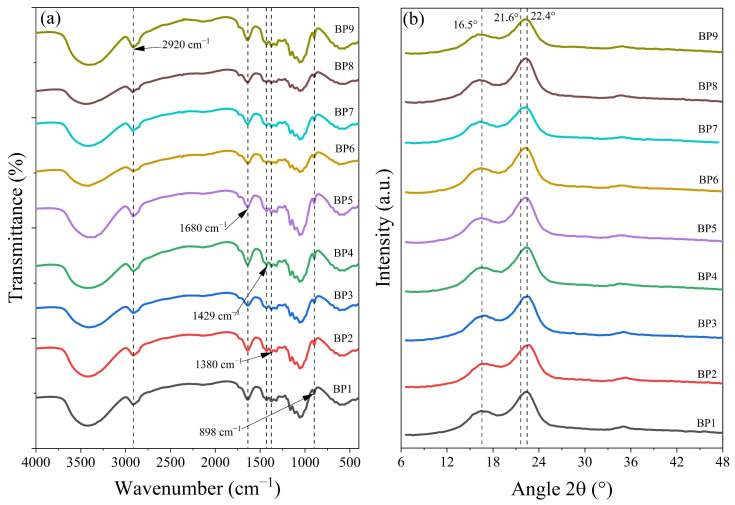
FTIR and XRD analyses of bleached pulps (BP1 to BP9) from unbleached pineapple crown pulp as a function of bleaching agent volume and reaction time. (**a**) FTIR spectra and (**b**) XRD patterns.

The FTIR spectra of the BPs (Figure 3a) showed significant structural changes when compared to the Ubp4 spectrum (Figure 2a). The disappearance of bands in 1735, 1509, and 1236 cm^−1^, typical of functional groups present in lignin and hemicelluloses, is observed, which corroborates the efficiency of delignification and purification of the cellulosic fraction obtained after alkaline extraction processes combined with bleaching of agricultural by-products [77].

The efficiency of delignification is also verified by the increase in peaks at 2920 and 1380 cm^−1^, corresponding to C−H deformation in pyranose rings [64]. The I_BP2920_ and I_BP1380_ values of the BPs were higher than those observed in Ubp4 (2.72 and 1.69, respectively), ranging from 4.25 (BP5) to 10.21 (BP9) for I_BP2920_, and from 1.21 (BP6) to 2.46 (BP5) for I_BP1380_ (Table 4). This increase reinforces the idea that the carbohydrate fraction becomes more accessible and concentrated in the pulp after bleaching.

The same samples that showed a reduction in I_BP1380_ also exhibited the largest decreases in I_BP898_ (amorphous cellulose), whose value ranged from 3.62 (Ubp4) to 2.76 (BP3), 2.09 (BP6), 2.85 (BP7), and 2.25 (BP8) (Table 4). This behavior suggests that larger clarifying solution volumes and/or prolonged times can lead to cellulose degradation, indicating a process limit beyond which losses in cellulosic fiber integrity may occur.

The band at 1429 cm^−1^, attributed to asymmetric CH_2_ deformation present in the crystalline regions of cellulose [78], confirmed the positive effect of the treatments on cellulose crystallinity. Samples BP4 (65 mL/60 min), BP5 (65 mL/90 min), and BP9 (75 mL/120 min) more efficiently preserved the cellulose structure, resulting in a higher cellulosic content after bleaching, with values close to those of Ubp4. On the other hand, BP6 (65 mL/120 min) and BP8 (75 mL/90 min) promoted a marked loss of crystallinity, corroborating the hypothesis that process severity must be controlled to avoid damage to the cellulose structure.

#### 3.2.2. X-Ray Diffraction Analysis of BP Samples

The I_BP(200)_, FWHM, and CrI_BP_ values ranged from 78.94 to 100.22 a.u., from 2.64 to 2.81 2θ, and from 49.42 to 59.11%, respectively (Table 4). These results indicate that bleaching conditions directly influence the structural organization of cellulose.

Except for BP1 (I_BP(200)_ = 78.84 a.u.) and BP3 (I_BP(200)_ = 82.26 a.u.), the bleaching process promoted greater structural organization of the crystalline region of the bleached pulps compared to Ubp4 (I_BP(200)_ = 82.61 a.u.). BP2, BP4, and BP5 achieved the highest I_BP(200)_ values (107.98 a.u.; 104.50 a.u.; and 106.78 a.u., respectively), which is attributed to the removal of residual lignin, which reduces the amorphous region and allows greater detection of the crystalline region [79].

FWHM is directly related to crystallite size and cellulose crystallinity [80]. Lower FWHM values indicate larger and more uniform crystallites, while broader peaks suggest a reduction in crystallite size. In Table 4, it is observed that, except for BP2 (FWHM = 2.64), all other samples showed a slight increase in FWHM between 2.75 and 2.81. BP2 stood out by simultaneously presenting the lowest FWHM and the highest I_BP(200)_ = 107.98 a.u. among all samples. This behavior indicates that bleaching with 55 mL/90 min promoted efficient removal of residual lignin without compromising the integrity of the crystalline domains, resulting in larger and more uniform crystallites, as well as greater exposure of the cellulose crystalline planes.

For BP3 to BP9, a simultaneous increase in I_BP(200)_ and FWHM was observed. This may be due to the removal of amorphous material (evidenced by the increased intensity), allowing the contribution of crystalline cellulose to be detected more clearly, and a slight peak broadening indicating that the alkaline swelling promoted by bleaching caused a small reduction in crystallite size or an increase in size heterogeneity, possibly due to the partial disorganization of crystalline regions [81].

ANOVA in Table 5, based on the applied polynomial model ($R_{adj.}^{2}$ = 0.999), confirmed a significant effect on cellulose crystallinity for the volume of the bleaching solution (*F* = 12,246.9; *p* < 0.001), reaction time (*F* = 6454.0; *p* < 0.001), and the interaction between both (*F* = 13,779.2; *p* < 0.001).

All interactions (V × t2, V × t2^2^, V^2^ × t2, V^2^ × t2^2^) were highly significant (*p* < 0.001) (Table 5), with emphasis on the V × t2^2^ interaction (*F* = 34112.14) and V^2^ × t2^2^ interaction (*F* = 16,285.38). This shows that the effect of one variable on CrI is strongly dependent on the level of the other, and that this dependence is complex, involving non-linear components. The variation in CrI_BP_ as a function of time follows a quadratic pattern for each volume tested. The representative mathematical model is:

CrI_BP_ = −2134.73 + 63.24 V − 0.45 V^2^ + 53.79 t2 − 0.31 t2^2 −^ 1.56 V × t2 + 0.01 V × t2^2^ + 0.01 V^2^ × t2 + 6.5 10^−5^ V^2^ × t2^2^ (5)

**Figure 4:**
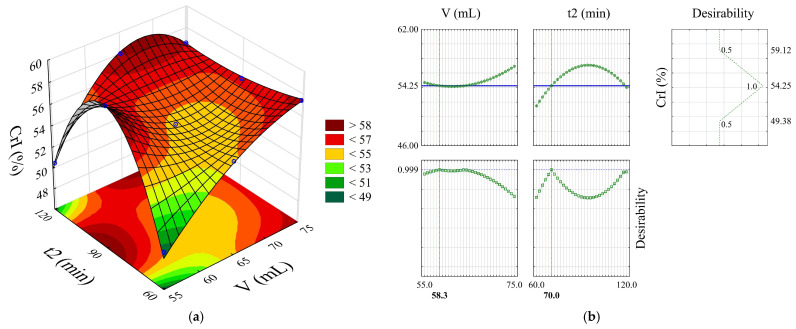
Crystallinity index (CrI) results of bleached pulp from unbleached pineapple crown pulp as a function of bleaching agent volume (V) and reaction time (t2). (**a**) Response surface plot and (**b**) desirability plot.

The region of highest response (red shades) on the response surface (Figure 4a) extends along a plateau, indicating operating conditions that favor high crystallinity values. The overall desirability (D = 0.999, Figure 4b) allowed the identification of the optimal point, using 65 mL of bleaching solution for 90 min (BP5), a condition under which a CrI_BP_ of 54.72% is predicted.

Based on the spectroscopic characteristics (FTIR and DRX) and the desirability value, BP5 was selected as the most suitable treatment for CMC synthesis, even though it did not present the highest CrI, observed in BP2. BP5 showed lower content of lignocellulosic impurities that could interfere with the carboxymethylation reaction (I_BP2920_ = 4.25); a good balance between crystallinity (CrI_BP_ = 54.73%) and purity, ensuring adequate reactivity without compromising CMC viscosity; intermediate and consistent values for the other parameters (I_BP(200)_ = 106.78 a.u.; FWHM = 2.75), evidencing good structural organization; and an intermediate reaction time (90 min) and moderate clarifying solution volume (65 mL), providing a better cost–benefit ratio in terms of reagent consumption and process time.

### 3.3. Structural and Thermal Analysis of CMCs

#### 3.3.1. Fourier Transform Infrared Spectroscopy Analysis of CMC Samples

**Figure 5:**
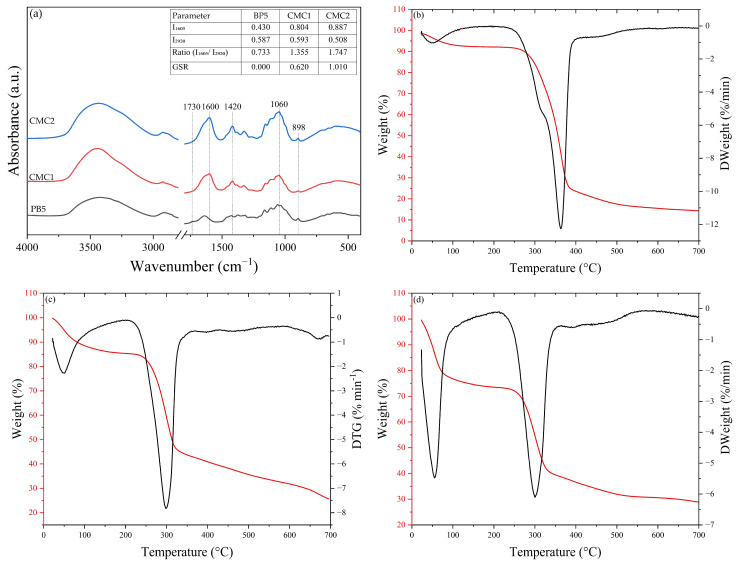
Spectrophotometric and thermogravimetric analyses of carboxymethylcelluloses and bleached pineapple crown pulp (BP5). (**a**) FTIR, (**b**–**d**) Thermogravimetry (–––) and derivative thermogravimetry (–––) curves of BP5, CMC1, and CMC2, respectively.

The introduction of the carboxymethyl group (COO^−^) into the cellulose structure is evidenced by the significant increase in the bands at 1600 and 1420 cm^−1^ [82,83] in the CMC1 and CMC2 spectra when compared to BP5 (Figure 5a). These bands increased, respectively, from 0.180 and 0.133 for BP5 to 0.452 and 0.621 for CMC1, and to 0.621 and 0.353 for CMC2, confirming the effectiveness of the carboxymethylation reaction, with effective incorporation of functional groups into the cellulose chain.

The band at 1730 cm^−1^, attributed to C=O stretching of carboxylic groups, showed low values in all samples (0.029–0.047), indicating that carboxylic groups are predominantly in the carboxylate salt form, which is desirable for water solubility and applications in films and coatings.

As discussed previously, the bands at 898 cm^−1^ (β-glycosidic bonds) and 1060 cm^−1^ (C–O–C stretching of the glycosidic ring) are indicators of cellulose structural integrity. CMC2 showed higher intensity at the 1060 cm^−1^ band (0.74) compared to BP5 (0.38), indicating greater structural preservation, while CMC1 (0.43) maintained a value close to that of BP5. The 898 cm^−1^ band remained present in all samples (Figure 5a), confirming that the polymeric structure of cellulose was preserved during the carboxymethylation process.

#### 3.3.2. Thermogravimetric Analysis of CMC Samples

The TGA and DTGA curves (Figure 5b–d) for BP5, CMC1 and CMC2 presented thermal degradation profiles that can be grouped into two different categories (Table 6).

BP5 presented two thermal degradation events and a significant reduction in carbonized residue, also observed in cellulosic pulps from pineapple crown [84]. Mass losses in the first event were 6.4%, while in the second event (cellulose decomposition) losses were 69.5%, resulting in an RC of 24.5%.

Unlike BP5, the CMCs showed distinct behavior. CMC1 exhibited water loss of 13.60% (Tmax = 50.23 °C), modified cellulose degradation of 38.10% (Tmax = 294.18 °C), and a third residual carbonization event of 22.50%, with a final RC of 25.60%. CMC2, on the other hand, presented a third degradation event and higher RC. During thermal degradation, the first event (water loss) was 25.0% (Tmax = 51.54 °C), the second event (modified cellulose degradation) was 34.50% (Tmax = 298.85 °C), and the third event (carboxymethyl group carbonization) was 11.20%, resulting in a final RC of 28.90%. These results corroborate data reported in the literature for pineapple-derived materials [85,86].

The second degradation event observed in the CMC thermograms occurred in the range of 235–340 °C, associated with the decomposition of carboxymethyl groups and the modified cellulose chain. This process resulted in reduced thermal stability, with temperatures of 294.18 °C (CMC1) and 298.85 °C (CMC2), compared to 363.94 °C for BP5 (Table 6). This decrease indicates that the introduction of COO^−^ disorganizes the crystalline structure of cellulose, making it more susceptible to thermal degradation.

The RC values increased in the CMCs, 25.60% and 28.90% for CMC1 and CMC2, respectively, higher than that of BP5 (24.50%), attributed to the presence of carboxymethyl groups that form sodium salts (COO^−^Na^+^) during pyrolysis, remaining after degradation of the organic matrix. The greater number of incorporated carboxymethyl groups was due to the more drastic synthesis conditions of CMC2 (50% NaOH, 7 g chloroacetic acid, 2.5 h) than those of CMC1 (30% NaOH, 5 g, 2.0 h). The higher RC of CMC2 compared to CMC1 suggests a higher degree of substitution (GSR, Figure 5a). This result is consistent with the FTIR analyses, in which CMC2 showed higher I_1605_ (0.887) and lower I_1920_ (0.508), resulting in an I_1605_/I_1920_ ratio of 1.747 and GSR = 1.010, 1.62 times higher than that of CMC1 (GSR = 0.620) (Figure 5a).

Although a higher GSR suggests lower thermal stability due to greater structural disorganization of cellulose [87], CMC2 presents a higher maximum degradation temperature (298 °C) compared to CMC1 (294 °C). However, the greater mass loss in event I for CMC2 (25%) compared to CMC1 (13%) indicates higher hygroscopicity, directly related to the more drastic synthesis conditions of CMC2, which resulted in a higher degree of substitution and, consequently, greater incorporation of hydrophilic carboxymethyl groups capable of retaining more water through hydrogen bonding and electrostatic interactions.

GSR is an important parameter for coating applications, as it directly influences water solubility, film-forming capacity, and barrier properties. In this context, CMC2, having a higher GSR, may form denser and more homogeneous films with better adhesion to the seed surface.

### 3.4. Effect of CMC Associated with B. subtilis on Common Bean Seed Coating

The physiological quality of common bean seeds coated with CMC (associated or not with *B. subtilis*) was evaluated by the parameters first count (FC), germination (GER), seedling length (SL), and seedling dry mass (SDM). The mean values ± standard deviation and the ANOVA results (mean square, *F* and *p* tests) are presented in Table 7.

The untreated control seeds (control) exhibited high germination (90.67%) and first count (82.00%), which were statistically similar (p > 0.05) to the best-performing treatments, CMC1 and C1BS (91.00% and 90.33% for germination, respectively). This could raise the question of whether the intrinsic quality of the bean species or seed lot masked potential treatment effects. However, the high physiological quality of the seed lot used (*P. vulgaris* L., cultivar BRS FC104) is methodologically desirable, as it provides a reliable baseline for detecting both negative and neutral effects of the coating formulations.

Except for the CMC2 treatment, which showed germination of 77.67% (below the legal standard of 80%), all other treatments—control, CMC1, BS, and C1BS—achieved germination percentages within the required range for commercialization [88]. For the C2BS treatment, it was not possible to determine final germination, as the seeds showed fungal contamination during the test, probably due to the excess moisture retained by the high degree of substitution of CMC2. However, some seeds in this treatment germinated sufficiently to allow evaluation of seedling length (18.37 cm) and dry mass (0.262 g), as shown in Table 7.

Unlike SDM, whose ANOVA test was not significant (*F* = 0.848, *p* = 0.527), Table 7 reveals significant differences among treatments for GER, FC, and SL (7.0 < *F* < 252.0, *p* < 0.001). The results demonstrate that CMC2 negatively affected common bean seed quality, significantly reducing the percentages of GER and FC (77.67% and 72.67%, respectively) compared to the control and to treatments with CMC1, whether associated or not with *B. subtilis*. This negative effect probably stems from the high water absorption capacity of CMC2 due to its high degree of substitution. The greater number of carboxymethyl groups in the polymer chain forms a more hydrophilic and expanded gel than CMC1, generating a coating layer with greater water retention. Excess moisture around the seeds created favorable conditions for the proliferation of opportunistic fungi, resulting in visible contamination (mold) and impaired seed viability. This fact is consistent with the lower FC (72.67%) and GER (77.67%) percentages observed in the CMC2-only treatment compared to the control (82.00% and 90.67%, respectively) and corroborates recent results in wheat seed coating with CMC, in which moderate polymer concentrations (0.25–0.5%) improved germination and vigor by approximately 5–10%, while higher concentrations reduced growth, probably due to excessive rigidity of the film formed around the seed [89].

On the other hand, CMC1 (lower degree of substitution) showed less swelling and did not induce critical moisture levels around the seeds, maintaining germination at levels equivalent to the control (91.00%). When associated with *B. subtilis* (C1BS), an increase in seedling length (21.30 cm) was observed, suggesting that the microorganism may have efficiently colonized the rhizosphere without being harmed by excess water.

The C2BS association, although not evaluated for germination parameters (missing data), showed seedling length (18.37 cm) and dry mass (0.262 g) higher than CMC2 alone. This indicates that *B. subtilis* may have acted as a biocontrol agent, reducing the incidence of filamentous fungi that benefits from the excess moisture provided by CMC2. However, the absence of germination data for this treatment limits the statistical confirmation of this hypothesis, and complementary studies with direct microbiological evaluation (counting of colony-forming units of fungi and bacteria on coated seeds) are necessary.

The contrasting results between CMC1 and CMC2 suggest the existence of a limit for the degree of substitution and/or polymer viscosity in seed coating applications. While CMC1 (lower DS) showed performance compatible with the control and, when associated with *B. subtilis*, promoted a significant increase in seedling length (21.30 cm), CMC2 exceeded this limit, resulting in indirect phytotoxicity due to microbial proliferation.

Given the above, the data demonstrate that CMC synthesized from pineapple crown, provided that the degree of substitution is controlled (CMC1), presents promising potential as a coating agent for common bean seeds, and can be used alone or as a vehicle for *B. subtilis*, without compromising germination and with additional vigor gains. This result is particularly relevant from the perspective of sustainable use of agro-industrial waste, since the pineapple crown—discarded as waste—proved to be a viable raw material for obtaining a functional polymer for agricultural applications. Therefore, the data indicates that the degree of substitution of CMC is a critical parameter to be optimized in the development of seed coating formulations. Formulations with high DS, although potentially more hydrophilic, can be counterproductive, especially for seeds with more sensitive coats, such as common beans. It is recommended that future studies evaluate intermediate DS values or adjust polymer concentration to balance the benefits of protection and microorganism delivery without harming germination processes.

## 4. Conclusions

The optimization of cellulosic pulp production from pineapple crown demonstrated that moderate conditions (2% NaOH/1.5 h) are as effective as severe conditions for delignification, achieving a Kappa number of 8.3 and CrI of 76.35%, with preservation of cellulose type I and accessible residual lignin. The optimized bleaching (65 mL/90 min) resulted in pulp with a CrI of 54.73%, suitable for carboxymethylation. CMC synthesis under different conditions produced materials with distinct degrees of substitution: CMC1 (moderate DS) and CMC2 (high DS), confirmed by FTIR and TGA.

In the evaluation as a coating agent for common bean seeds, CMC1 did not compromise germination (91.0%) and, when associated with *B. subtilis*, promoted a significant increase in seedling length (21.30 cm). In contrast, CMC2 negatively affected germination (77.67%) due to its higher hydrophilicity, which resulted in excessive water retention, fungal proliferation, and impaired seed viability.

In conclusion, the DS of CMC is a critical parameter for seed coating applications and should be controlled to avoid indirect phytotoxic effects. The pineapple crown proves to be a sustainable and viable raw material for producing CMC with potential agricultural use, particularly when synthesized with moderate DS.

## Figures and Tables

**Table 1:** Mean values and standard deviations of Kappa number (*κ*) and delignification efficiency (E*κ*) of pineapple crown biomass (PCB) obtained from a full 3^2^ factorial design for NaOH concentration (C1) and reaction time (t1).

Treatments	C1 (%)	t1 (h)	*κ*	E*κ* (%)
PCB	-	-	51.5 ± 2.2	-
Ubp1	1.0	1.5	21.8 ± 1.7	57.67
Ubp2	1.0	2.0	17.8 ± 0.3	65.44
Ubp3	1.0	2.5	17.2 ± 1.6	66.60
Ubp4	2.0	1.5	8.3 ± 0.3	83.88
Ubp5	2.0	2.0	12.1 ± 0.3	76.50
Ubp6	2.0	2.5	16.9 ± 1.3	67.18
Ubp7	3.0	1.5	15.6 ± 0.7	69.71
Ubp8	3.0	2.0	9.5 ± 0.3	81.55
Ubp9	3.0	2.5	8.1 ± 0.4	84.27

- Not determined. Ubp1 to Ubp9—unbleached pulp.

**Table 2:** Analysis of variance for Kappa number, regression coefficients (CR_Ubp_) and *t*-test (*t*) results as a function of NaOH concentration (C1) and reaction time (t1) for pineapple crown biomass obtained from a full 3^2^ factorial design.

Factor	df	MS	*F*	*p*	CR_Ubp_	*t*
C1_LQ_ (%)	2	159.04	174.80 **	<0.001	-	-
t1_LQ_ (h)	2	9.94	10.93 **	<0.001	-	-
C1_LQ_ × t1_LQ_	4	56.17	61.74 **	<0.001	-	-
Mean/Interaction	-	-	-	-	216.39 **	4.74
C1	1	277.32	304.79 **	<0.001	−216.95 **	−4.19
C1^2^	1	40.76	44.80 **	<0.001	55.19 **	4.31
t1	1	5.56	6.11 *	0.020	−142.64 **	−3.02
t1^2^	1	14.32	15.74 **	<0.001	23.89 ^n.s.^	2.03
C1t1	1	6.35	6.98 *	0.017	149.26*	2.79
C1 × t1^2^	1	0.39	0.44 ^n.s.^	0.518	−23.02 ^n.s.^	−1.72
C1^2^ × t1	1	214.86	236.15 **	<0.001	−38.94 **	−2.94
C1^2^ × t1^2^	1	3.07	3.37 ^n.s.^	0.083	6.07 ^n.s.^	1.84
Error	18	0.9098	-	-	-	-
$R_{adj.}^{2}$ = 0.958						

C1_LQ_ and t1_LQ_—global factors of concentration and time, respectively. L and Q in C1 and t1—linear and quadratic components. df—degrees of freedom. MS—mean square. *F*—F-test. *p*—probability value. *, **—Significance at the 1% an 5% probability levels, respectively. ^n.s.^ = not significant. - Not determined.

**Table 3:** Peak intensities of the main FTIR bands and crystallinity parameters from X-ray diffraction of unbleached pulps from pineapple crown biomass obtained from a full 3^2^ factorial design for NaOH concentration (%) and reaction time (h).

Treatments	Wavenumber (cm^−1^)							Crystallinity Parameters	
	I_2920_	I_2885_	I_1730_	I_1630_	I_1380_	I_1250_	I_898_	I_(200)_ (u.a.)	CrI_Ubp_ (%)
PCB	5.18	0.17	2.09	11.77	1.36	2.4	0.33	39.29	59.30
Ubp1 (1%/1.5 h)	2.74	0.03	0.00	9.4	1.83	0.33	4.22	82.39	79.77
Ubp2 (1%/2.0 h)	2.69	0.06	0.00	5.9	3.32	0.51	2.58	85.66	64.69
Ubp3 (1%/2.5 h)	3.03	0.01	0.00	6.67	1.67	0.50	2.94	70.81	75.98
Ubp 4 (2%/1.5 h)	2.72	0.01	0.00	5.92	1.69	0.61	3.62	88.47	76.35
Ubp 5 (2%/2.0 h)	2.18	0.00	0.00	4.23	1.87	0.50	3.23	80.68	80.29
Ubp6 (2%/2.5 h)	2.12	0.00	1.26	4.36	1.59	0.19	2.41	75.07	75.43
Ubp7 (3%/1.5 h)	3.55	0.06	0.00	4.79	1.75	0.22	2.36	92.18	79.53
Ubp8 (3%/2.0 h)	4.43	0.00	0.00	7.11	1.65	0.34	2.44	77.29	89.25
Ubp9 (3%/2.5 h)	2.05	0.02	0.00	3.7	1.63	0.33	2.85	84.01	82.17

PCB—pineapple crown biomass. Ubp1 to Ubp9—unbleached pulps. I_2920_, …, I_898_—corresponding peak intensities at bands 2920 cm^−1^, …, 898 cm^−1^ in FTIR. I_(200)_—peak intensity at plane (200). a.u.—arbitrary unit. CrI_Ubp_—crystallinity index.

**Table 4:** Peak intensities of the main FTIR bands and crystallinity parameters from X-ray diffraction of bleached pulps (BP1 to BP9) from unbleached pineapple crown pulp (Ubp4) obtained from a full 3^2^ factorial design for bleaching agent volume (V) and reaction time (t2).

Variable	Wavenumber (cm^−1^)					X-Ray Diffraction Parameters		
	I_BP2920_	I_BP1630_	I_BP1429_	I_BP1380_	I_BP898_	I_BP(200)_ (u.a.)	FWHM (2θ)	CrI_BP_ (%)
Ubp4	-	-	-	-	-	82.61	2.72	76.35
BP1 (V= 55 mL, t2= 60 min)	6.14	11.00	2.17	1.88	3.60	78.94	2.72	49.42
BP2 (V= 55 mL, t2= 90 min)	7.23	7.71	2.17	1.88	3.62	107.98	2.64	59.11
BP3 (V= 55 mL, t2= 120 min)	6.21	8.11	1.63	1.55	2.76	82.26	2.80	50.50
BP4 (V= 65 mL, t2= 60 min)	6.54	7.00	2.44	1.96	4.45	104.50	2.72	54.73
BP5 (V= 65 mL, t2= 90 min)	4.25	9.94	2.59	2.45	4.25	106.78	2.75	54.73
BP6 (V= 65 mL, t2= 120 min)	4.28	11.33	1.24	1.21	2.09	101.46	2.78	58.09
BP7 (V= 75 mL, t2= 60 min)	7.01	6.63	1.71	1.64	2.85	101.07	2.75	57.46
BP8 (V= 75 mL, t2= 90 min)	5.65	10.52	0.83	1.33	2.25	95.03	2.81	56.32
BP9 (V= 75 mL, t2= 120 min)	10.21	10.78	2.39	2.18	4.42	100.22	2.78	56.60

I_BP2920_, …, I_BP898_—corresponding peak intensities at bands 2920 cm^−1^, …, 898 cm^−1^ in FTIR. I_BP(200)_—intensity of the crystalline peak corresponding to the (200) plane (22.5° 2θ). FWHM—full width at half maximum. CrI_BP_—crystallinity index. - Not determined.

**Table 5:** Analysis of variance, regression coefficient (CR_BP_), and adjusted coefficient of determination ($R_{adj.}^{2}$) of the polynomial regression model fitted to the crystallinity index values of the bleaching process of unbleached pulp obtained from pineapple crown biomass, as a function of bleaching agent volume (V) and reaction time (t2).

Factor	df	MS	*F*	*p*	CR_BP_	*t*
V_LQ_	2	23.27	12,246.89	<0.01	-	-
t2_LQ_	2	12.26	6454.00	<0.01	-	-
V_LQ_ × t2_LQ_	4	26.18	13,779.23	<0.01	-	-
Interception	-	-	-	<0.01	−2134.73	−129.72
V	1	42.94	22,600.44	<0.01	63.24	123.30
V^2^	1	3.60	1893.34	<0.01	−0.45	−114.75
t2	1	4.26	2242.21	<0.01	53.79	138.69
t2^2^	1	20.27	10,665.79	<0.01	−0.31	−144.86
V × t2	1	1.88	990.42	<0.01	−1.56	−129.35
V × t2^2^	1	64.81	34,112.14	<0.01	0.01	135.68
V^2^ × t2	1	7.09	3728.98	<0.01	0.01	121.18
V^2^ × t2^2^	1	30.94	16,285.38	<0.01	6.5 10^−5^	−127.61
Error	9	0.002	-	-	-	-
$R_{adj.}^{2}$ = 0.999						

V_LQ_ and t2_LQ_—global factors of volume and reaction time, respectively. L and Q in V and t2—linear and quadratic components. df—degrees of freedom. MS—mean square. *F*—F test. *p*—probability value. *t*—t-test.

**Table 6:** Thermogravimetric analysis (TGA and DTGA) parameters for bleached pineapple crown pulp (BP5) and carboxymethylcellulose (CMC1 and CMC2).

Sample	Event	Ti (°C)	Te (°C)	Tf (°C)	Tmax (°C)	Δm (%)	RC (%)
BP5	I	25.00	30.26	125.72	53.35	6.40	24.50
	II	231.44	267.65	419.97	363.94	69.50	
CMC1	I	22.71	26.52	119.84	50.23	13.60	25.60
	II	204.00	239.89	325.18	294.18	38.10	
	III	~400	~400	688.30	673.57	22.50	
CMC2	I	23.52	23.52	151.64	51.54	25.00	28.90
	II	211.48	235.01	338.10	298.85	34.5	
	III	~400	~400	698.01	597.86	11.20	

Ti—initial temperature. Tf—final temperature. Te—onset temperature. Tmax—maximum degradation temperature. Δm—percentage of sample mass loss. RC—carbonization residue.

**Table 7:** Mean values ± standard deviation, ANOVA summary, and mean test for physiological quality tests of common bean seeds coated with carboxymethylcellulose from pineapple crown pulp associated or not with *Bacillus subtilis*.

Treatments	Germination (%)	First Count (%)	Seedling Length (cm)	Seedling Dry Mass (g)
Control	90.67 ± 4.13 a	82.00 ± 6.93 b	15.32 ± 4.76 bc	0.197 ± 0.027 a
CMC1	91.00 ± 8.74 a	83.00 ± 8.56 b	12.63 ± 1.87 c	0.215 ± 0.012 a
CMC2	77.67 ± 6.38 b	72.67 ± 3.93 c	13.22 ± 2.31 bc	0.218 ± 0.028 a
*B. Subtillis* (BS)	89.00 ± 4.86 a	82.67 ± 1.63 b	14.30 ± 3.05 bc	0.295 ± 0.044 a
CMC1 + *B. Subtillis* (C1BS)	90.33 ± 5.43 a	89.33 ± 5.01 a	21.30 ± 2.88 a	0.188 ± 0.029 a
CMC2 + *B. Subtillis* (C2BS)	0.00 ± 0.00 c	0.00 ± 0.00 d	18.37 ± 2.84 ab	0.262 ± 0.061 a
ANOVA	Physiological quality tests			
	Germination (%)	First count (%)	Seedling length (cm)	Seedling dry mass (g)
Mean square	6883.80	7851.90	67.18	0.001
*F*	251.24	251.66	7.049	0.848
*p*	<0.001	<0.001	<0.001	0.527

## Data Availability

The original contributions presented in this study are included in the article. Further inquiries can be directed at the corresponding author.
